# Data on COVID-19-related Research Questions Spanning Diverse Disciplinary and National Contexts

**DOI:** 10.5334/jopd.111

**Published:** 2024-04-08

**Authors:** Katarina Blask, Débora B. Maehler, Martin Kerwer

**Affiliations:** 1 Leibniz Institute for Psychology (ZPID), DE; 2 GESIS –Leibniz-Institute for the Social Sciences, DE

**Keywords:** COVID-19, research data, quantitative studies, socio-political attitudes, conspiracy theories

## Abstract

The special collection “Data for Psychological Research on COVID-19” presents selected datasets collected during the COVID-19 pandemic. In selecting the data papers, care was taken to ensure that the described datasets not only represent the full range of psychological topics addressed during the pandemic but also reflect its global nature, in that diverse nationalities were included in the investigated samples. As these datasets are shared according to the FAIR Principles (Wilkinson et al., 2016), they are findable, accessible, interoperable and reusable. The special collection comprises 12 data papers presenting quantitative studies on the impact of COVID-19 on various psychological constructs, including socio-political attitudes, beliefs in conspiracy theories, emotional reactions, and control behaviour.

The first cases of the pulmonary disease COVID-19 were observed in Wuhan, People‘s Republic of China in December 2019. Within a very short time, the disease had become a global pandemic with many devastating consequences. For example, according to the World Health Organization (WHO, 2023), over 6.8 million COVID-related deaths have been recorded since 2019. However, the pandemic – and especially the need to rapidly develop suitable vaccines and therapeutic strategies – also had some positive side effects. In particular, it resulted in a tremendous surge in research to fight the new coronavirus, and in funding for this research. In 2020, the European Commission pledged €1 billion from the EU’s Horizon 2020 programme for research and innovation projects to fight the pandemic. In late 2019, the U.S. National Institutes of Health (NIH) developed a NIH-Wide Strategic Plan for COVID-19 Research, which was guided by five strategic priorities: (1) Improve our knowledge about the disease; (2) Develop diagnostic tests; (3) Discover and develop treatments; 4) Develop vaccines to improve protection and prevention; (5) Understand the risk to and provide support for people at higher risk of infection (NIH, n.d.). While the first four priorities were addressed mainly by medical research initiatives, Priority 5 was also addressed by a vast number of studies in psychology conducted during the pandemic with the aim of better understanding psychosocial factors related to COVID-19 and its influence on human behaviour and experience (e.g., Van Bavel et al., 2020). Moreover, to join forces in the fight against the pandemic and to create a common knowledge base, researchers in psychology and related disciplines published not only more preprints but also data and code related to their research (see Besançon et al., 2021).

Easy access to and widespread dissemination of this knowledge was of the utmost importance for the management of the pandemic and its impacts (Van Bavel et al., 2020). For instance, it helped to gain a better understanding of people’s reactions to the pandemic and to public health measures to fight it. It could also provide valuable insights into people’s reactions to global threats and their coping strategies to counter these threats. More specific topics addressed by researchers within this broader context range from moral decision-making and cooperation behaviour, through the impact of conspiracy theories and fake news, to the investigation of interindividual differences, for example, regarding culture, political attitudes and social background. To ensure the widest possible dissemination of the findings from COVID-19 research, various measures were taken by the research communities and related infrastructure and service providers. These measures ranged from collaborative data publication initiatives such as the COVID-19 Open Research Dataset (CORD-19; Wang et al., 2020) and the OpenSAFELY analytics platform (OpenSafely.org, n.d.) to reduced reviewing times for manuscripts reporting the findings from COVID-19-related research (i.e. fast-tracking of COVID-19 papers; see also Besançon et al., 2021). However, although all these measures contributed to the rapid dissemination of and easy access to the knowledge, the fast-tracking of COVID-19-related papers also resulted in the dissemination of unqualified knowledge in some cases (Besançon et al., 2021).

In addition to well established formats such as preregistration, innovative publication formats to counter the dissemination of unqualified knowledge and increase the reusability of research gained traction and were broadly used by researchers in psychology and related disciplines. They include, for example, data papers, which are a very good way to add context to data and thus increase their reuse potential. Data papers are the key publication format in the *Journal of Open Psychology Data*, whose editorial board comprises not only researchers from psychology, but also data experts who work, for example, at established data infrastructures. This ensures not only that researchers’ data papers are reviewed for their potential contribution to research in the field and for the excellence of the methodology used to collect the data, but also that the data and associated documentation undergo comprehensive quality review.

The present special collection, “Data for Psychological Research on COVID-19”, makes use of this innovative publication format to increase awareness of selected datasets collected during the COVID-19 pandemic. In selecting the data papers, care was taken to ensure that the described datasets not only represent the full range of psychological topics addressed during the pandemic but also reflect its global nature, in that diverse nationalities were included in the investigated samples. As these datasets are shared according to the FAIR Principles (Wilkinson et al., 2016), they are findable, accessible, interoperable and reusable. The special collection comprises 12 data papers presenting quantitative studies on the impact of COVID-19 on various psychological constructs, including socio-political attitudes, beliefs in conspiracy theories, emotional reactions, and control behaviour. For an overview of the topics and the nations represented in the samples, see Table 1.

**Table 1:** Overview of COVID-19 datasets: Design features and sample characteristics.


AUTHORS	TITLE	COUNTRY	SAMPLE	*N*	AGE (*M*)	SURVEY PERIOD	DESIGN	DATA ACCESS

Huber et al., 2023	The Relation Between the Public Attitude Towards COVID-19 and its Applied Policies: A Dataset for Binational and Temporal Comparison	Germany (DE); Switzerland (CH)	Adults	DE: 131 CH: 130	DE: 32; CH: 36	July 2020– May 2021	Cross-sectional; country comparison	10.5334/jopd.84

Abadi etal., 2023	A Dataset of Social-Psychological and Emotional Reactions During the COVID-19 Pandemic Across our European Countries	Germany, the Netherlands (NL), Spain (ES), and the UK	Adults	DE: 524; NL: 496; ES: 503; UK: 508	41	April 2020	Cross-sectional; country comparison	10.5334/jopd.86

Reim et al., 2022	Data from the German Family Panel Pairfam: The Supplementary COVID-19 Survey	Germany	Adults	3,182	31	May–July 2020	Cross-sectional; longitudinal	10.5334/jopd.68

Edlund & Edlund, 2022	COVID-19 and Psychology: Questionnaire Data From Two SCORE Projects	USA	Adults	167	35	August 2020	Cross-sectional	10.5334/jopd.66

Hudecek et al., 2022	Who Thinks COVID-19 is a Hoax? Psychological Correlates of Beliefs in Conspiracy Theories and Attitudes Towards Anti-Coronavirus Measures at the End of the First Lockdown in Germany	Germany	Young adults	746	26	May–July 2020	Cross-sectional	10.5334/jopd.64

Ingramet al., 2022	Mental, Physical, and Cognitive Wellbeing during the COVID-19 Pandemic: Data from Scotland and Japan	Scotland; Japan	Adults	Study 1: 399 Study 2: 277	Study 1: 32 Study 2: 39	May 2020; February 2021	Cross-sectional; country comparison	10.5334/jopd.65

Parsons et al., 2022	Data and protocol for the Oxford Achieving Resilience During COVID-19 (ARC) Study	UK	Adolescents, adults	Children: 1,196 Parents: 464	Children: 15; Parents: 47	March 2020– August 2021	Longitudinal	10.5334/jopd.56

Reinelt et al., 2022	Survey and 10-Day Diary Data on Infant Nutrition, Development, and Home Learning Environment during the COVID-19 Pandemic from the LEARN-COVID Pilot Study	Austria, Germany, Switzerland	Adults	222	35	April–July 2021	Cross-sectional; longitudinal	10.5334/jopd.63

Ryvkina et al., 2023	Understanding Psychological Responses to the COVID-19 Pandemic Through ESM Data: The EMOTIONS Project	Germany	(Young) Adults	Study 1: 370 Study 2: 3,565	Study 1: 56 Study 2: 25	January–June 2020	Cross-sectional; longitudinal	10.5334/jopd.83

Schnepf & Groeben, 2022	The COVID-19 framing dataset: How Secondary Data Can Be Used to Explore Paradoxical Attitudes During the COVID-19 pandemic	Austria (AT), Germany, UK, and USA	Adults	5 samples: USA: 430; DE: 476; DE: 217, AT: 211; UK: 211	US: 37; DE: 46; DE: 43; A: 43; UK: 42	July 2020 to February 2021	Cross-sectional; country comparison	10.5334/jopd.62

Weiß & Stadtmüller, 2023	Using the probability-based GESIS Panel for longitudinal psychological research on the COVID-19 outbreak in Germany	Germany	Adults	4,786	57	May 2020 to July 2022	Cross-sectional; longitudinal	10.5334/jopd.90

Welzel et al., 2023	The Values in Crisis (ViC) Project: A two-wave panel study in Germany and the United Kingdom	Germany; UK	Adults	DE: 2,009; UK: 2,033	16–65	April 2020– April 2022	Longitudinal	10.4232/1.14148


The data papers in this special collection span the period from January 2020 to April 2022. Although the majority of the data were collected in German-speaking countries, they also include samples from the United Kingdom, the United States, Spain, the Netherlands, and Japan. The special collection brings together papers on a wide range of behaviours and psychological processes, focusing, for example, on compliance with and attitudes towards preventive measures or on the consequences of the COVID-19 pandemic for various psychological outcomes. For instance, some of the reported datasets address the attitudes of individuals in general population samples towards preventive public health measures such as mask mandates and vaccination, their endorsement of policy strictness (Huber et al., 2023; Weiß & Stadtmüller, 2023), and their social-psychological and emotional responses to these restrictions (Abadi et al., 2023). The dataset described in Parsons et al. (2022) is based on a more narrowly defined population – dyads of adolescents and their parents – and addresses psychological responses to COVID-19, such as resilience and anxiety. Further data papers describe datasets that cover the consequences of COVID-19 restrictions for family life and the well-being of parents and children at a more general level (Reim et al., 2022), or that focus on specific behavioural consequences, such as parental behaviour with regard to infant nutrition and infant regulation (Reinelt et al., 2022). The datasets described in other papers address the effects of COVID-19 restrictions on health behaviours and mental well-being (Ingram et al., 2022); widely discussed interindividual factors such as the endorsement of these restrictions, (dis)trust in science, and conspiracy mentality (Schnepf & Groeben, 2022); and psychological correlates of beliefs in conspiracy theories (Hudecek et al., 2022). Edlund and Edlund (2023) further strengthen this area of research by contributing data from replication studies on the effects of empathy and conspiracy beliefs on preventive health behaviours (see discussion above on the dissemination of unqualified knowledge). Finally, six contributions to this special collection describe longitudinal datasets (Parsons et al., 2022; Reinelt et al., 2022; Reim et al., 2022; Ryvkina et al., 2023; Weiß & Stadtmüller, 2023; Welzel et al., 2024) addressing, for example, people’s moral values during and shortly after the pandemic in Germany and the UK (Welzel et al., 2024) and – more generally – people’s emotional and behavioural states during and after the first nationwide COVID-19 lockdown in Germany (Ryvkina et al., 2023).

Looking at the bigger picture, it is important to bear in mind that all the COVID-19-related datasets presented in this special collection were collected under exceptional circumstances and at a unique point in time. From the perspective of future research, the COVID-19 pandemic could be seen as a natural quasi-experiment whose data collection cannot be reproduced or replicated. Preserving psychological data on the COVID-19 pandemic for future researchers is therefore of paramount importance. This could allow psychological science to inform policy and practice to improve future public health responses (see Collins et al., 2023, for a biomedical research perspective on this issue). In addition, the data presented in this special issue may also prove to be valuable in contexts that cannot be foreseen today. For example, psychological measurement instruments may function differently under pandemic conditions, or pandemic effects on specific psychological outcome variables may become relevant in future disaster management scenarios.

In summary, although the data papers in this special collection address a wide variety of psychological topics, they provide only a small glimpse at the tip of the iceberg formed by the vast amount of psychological research conducted during the COVID-19 pandemic. Therefore, we would like to encourage more psychological researchers to follow the example of the authors who contributed to this collection and to make their data on the COVID-19 pandemic available in a public repository.
